# A Multi-epitope Subunit Vaccine Identification and Development Against Scrub Typhus (Orientia tsutsugamushi) Using Immunoinformatics Approaches

**DOI:** 10.7759/cureus.61009

**Published:** 2024-05-24

**Authors:** Shalini Khichi, Sikha Morang, Puneet Dhamija, Shailendra Handu

**Affiliations:** 1 Pharmacology, All India Institute of Medical Sciences, Rishikesh, IND

**Keywords:** in silico cloning, molecular dynamics, molecular docking, multi-epitope vaccine, immunoinformatic, orientia tsutsugamushi

## Abstract

Background

The pathogen *Orientia tsutsugamushi*, which causes scrub typhus, is rapidly spreading throughout the tropics. As a measure to improve public health, the development of a vaccine for human use is essential. Scrub typhus is listed as one of the underdiagnosed and underreported febrile infections. This vector-borne zoonotic infection appears as eschar on the patient's skin.

Methods

Immunoinformatics was employed to predict the multi-epitope subunit vaccine that will activate both B and T cells. The final vaccine includes lipoprotein LprA as an adjuvant at the N-terminus along with B-cell, helper T lymphocyte (HTL), and cytotoxic T lymphocyte (CTL)-binding epitopes to boost immunogenicity. Assessing the vaccine's physiochemistry demonstrates that it is both antigenic and non-allergic.

The vaccine structure was developed, enhanced, confirmed, and disulfide-engineered to provide the best possible model. Using molecular docking, the interaction of the produced vaccine with toll-like receptor 2 (TLR2) was analyzed, and the vaccine-receptor complex was stabilized by molecular dynamics (MD) simulation. According to in silico cloning, *Escherichia coli* can efficiently produce the recommended vaccine. Additionally, the efficacy of the in silico-developed vaccine must be evaluated in an in vitro and in vivo experiment.

Results

The developed vaccine successfully stimulates cellular and humoral immune responses. The vaccine, which has three B-cell epitopes, three HCL epitopes, and nine CTL epitopes, can bind firmly to immunological receptors. Dynamic investigations of the vaccine-receptor complex show a strong interaction and stable conformation.

Conclusion

In this study, the vaccine candidate demonstrated strong antigenicity, stability, and solubility while also being non-allergenic to host cells. The vaccine candidate's stability with the TLR2 immune receptor is established by binding studies, and in silico cloning verifies efficient and stable expression in the bacterial system.

## Introduction

Scrub typhus is a vector-borne, zoonotic, infectious illness caused by *Orientia tsutsugamushi*, a gram-negative obligated intracellular bacterium of the *Trombiculidae* family. Small rodents are animal reservoirs for *O. tsutsugamushi*; however, the pathogen can also be sustained within mite colonies via transovarial transmission. *Orientia tsutsugamushi* is not as well-known as other infections with the same frequency and severity in terms of its basic cell biology [1]. Fever, rash, headache, nausea, vomiting, and thrombocytopenia are all typical symptoms that could be mistaken for malaria, chikungunya, dengue fever, enteric fever, leptospirosis, and brucellosis [2].

Scrub typhus is often observed in the Asia-Pacific region, which spreads from Pakistan, India, and Nepal in the west through south-eastern Siberia, China, Japan, and Korea in the north, and all the way to Indonesia. The Philippines and the southern Pacific islands are part of northern Australia. Cases of scrub typhus have been observed in the "tsutsugamushi triangle," the traditional endemic area. Globalization and greater travel have caused *Orientia tsutsugamushi* to spread from endemic to non-endemic areas of the world, resulting in a range of new strains with antigenic diversity [1,3].

Over the past 73 years, more than 15% of studies have used Gilliam, Karp, and Kato prototype strains as a baseline for developing improved diagnostics, therapies, and detection methods for scrub typhus occurrence. This highlights the importance of strains to understand the pathogenesis of scrub typhus [4]. The Karp strain was named the type strain of the new genus *Orientia*, while the Gilliam and Kato strains' 16S rRNA gene sequences served as the major molecular data used to distinguish scrub typhus strains from *Rickettsia* strains [5].

Only a few studies have been done on the most important antigenic types in our country. Therefore, knowing the types of antigens that are most common in India is important for the success of diagnostic immunoassays and possible vaccine candidates [6].

A set of infections known to cause neglected tropical diseases (NTDs) affects roughly 12.5% of the world's population. Scrub typhus is now being treated with antibiotics such as doxycycline, azithromycin, tetracycline, and rifampicin [7]. There are no commercial vaccines against *Orientia tsutsugamushi* yet, and those made from released bacteria (initial bodies) have a limited value due to the pathogen's extensive antigenic diversity, whilst those made from live attenuated organisms risk co-transmission of other blood-borne infections [8].

In comparison to vaccines made using traditional vaccinology, epitope-based chimeric/subunit vaccines offer several benefits. For example, they can replace many wet lab studies and save time because they are less expensive to manufacture and do not require microbial cultivation. They are a safer alternative since they are highly specific and stable and do not contain full viruses and bacteria [9].

A multi-epitope-based vaccination was made possible by recent improvements in computational techniques and immunoinformatics tools [10]. The current work screens three antigenic proteins to identify potential binders (cytotoxic T lymphocytes (CTL), helper T lymphocytes (HTL), and B-cell) using various immunoinformatics tools. After that, linkers were used to connect the chosen antigenic epitopes, and an adjuvant was included in the construct to increase its immunogenicity even further. The immunogenicity and allergenicity of the constructed vaccine were then predicted and further improved. Additionally, molecular docking and molecular dynamics studies were also executed to better understand the binding affinity and interaction patterns of the docked complex. Finally, in silico cloning was used to determine the efficacy and expression of the proposed vaccine.

## Materials and methods

Retrieval of protein sequences and physiochemical properties analysis

The protein sequence of *Orientia tsutsugamushi* (Gilliam strain) was retrieved from the Uniport (Universal Protein Resource) database (http://www.uniprot.org/uniprot) [11]. VaxiJen v 2.0 (http://www.ddg-pharmfac.net/vaxijen/VaxiJen/VaxiJen.html) [12] and Antigenic Peptide Prediction tool by the Immunomedicine Group (http://imed.med.ucm.es/Tools/antigenic.html) [13] server were used to predict the average antigenic propensities of the individual proteins.

In addition to this, the physiochemical properties of all proteins were identified by using the ExPASy server's ProtParam program (http://expasy.org/cgi-bin/protpraram) [14].

B-cell epitopes prediction

B-cell epitopes were predicted using a recurrent neural network-based server called ABCpred (http://crdd.osdd.net/raghava/abcpred/), which predicts B-cell epitopes (random peptides) up to 20 residues in length. For predicting B-cell lymphocytes (BCL), the server was trained on 700 B-cell epitopes and 700 non-B-cell epitopes. Using a recurrent neural network, this server's expected accuracy is 65.93% [15]. In the ABCpred server, the default BCL prediction threshold was set to 0.51. VaxiJen, AllerTOP, ToxinPred, and the TMHMM websites were used to screen anticipated B cell epitopes for selecting final candidates.

T-cell (CTL) epitopes prediction

NetCTL 1.2 server (http://www.cbs.dtu.dk/services/NetCTL/) was used to predict T cell epitopes. The server uses non-linear artificial neural networks to divide human leukocyte antigen (HLA) class I alleles into 12 super-families (A1, A2, A3, A24, A26, B7, B8, B27, B39, B44, B58, and B62) [16]. All three *Orientia tsutsugamushi* bacterial protein amino acid sequences were searched against A2, A3, and B7 HLA class I superfamily members (a total of 3 x 3 = 9 searches). The *Orientia tsutsugamushi* CTL prediction threshold values were 0.75. The epitopes that exceeded the threshold value were then chosen for further investigation. The antigenicity of the proteins was assessed using the VaxiJen server. Epitopes that failed to reach the 0.4 cutoff threshold set by the VaxiJen server were eliminated. The allergenicity of the chosen T-cell epitopes was also determined using AllerTOP. The epitopes' toxicity was also determined using the ToxinPred website. Additionally, to determine the anticipated epitopes transmembrane topology, the TMHMM server (http://www.cbs.dtu.dk/services/TMHMM/) was used.

T-cell (HTL) epitopes prediction

The IEDB MHC-II binding database (http://tools.iedb.org/mhcii/) was used to predict the human H-2-I allele binding epitopes from proteins. At the IEDB MHC-II binding interface, the IEDB-recommended technique was chosen as the prediction method, and a window length of 15 mer was chosen for CD4+ T cell (HTL) epitope prediction [17]. The web servers VaxiJen, AllerTOP, ToxinPred, and TMHMM were also used to examine these epitopes. After cross-checking with these servers, the generated epitopes were analyzed using the IFNepitope webserver (http://crdd.osdd.net/raghava/ifnepitope/). This server is used to predict interferon (IFN)-inducing epitopes from a library of peptides [18]. For the purpose of predicting IFN-inducing peptides, the amino acid sequences of all the previously chosen epitopes were utilized as a query. The majority of HLA class-II covering epitopes were chosen as final HTL epitopes and subjected to population coverage analysis, like CTL screening.

Toxicity prediction of the selected epitopes

Prior to developing the multi-epitope subunit vaccine, each of the chosen HTL, CTL, and BCL epitopes was individually assessed for its toxicity using the ToxinPred module (http://crdd.osdd.net/raghava/toxinpred/), which identifies toxic and non-toxic peptides supplied by a user [19].

Subunit vaccine construction using immunogenic epitopes

The multi-epitope vaccine was built using the highest-scoring BCL, CTL, and HTL epitopes, which were then linked together using the proper linkers. The most important function of linkers is to offer the amino acid residue the greatest degree of flexibility and to adequately separate the epitopes inside the human body. Using the EAAAK linker, an adjuvant with a sequence length of 244, known as lipoprotein LprA (P9WK55) agonist of toll-like receptor 2 (TLR2) was attached to the vaccine's (N-terminal) N-terminus. The HTL, CTL, and BCL epitopes were then joined to the linkers GPGPG, AAY, and KK to form the vaccine construct [20].

Prediction of various physicochemical properties

The ExPASy ProtParam program (http://web.expasy.org/protparam/), which evaluates based on the sequence and pKa values of the amino acids contained in the protein, was used to determine the physicochemical parameters of the vaccine construct. According to the "N-end rule," which states that a protein's in vivo half-life is related to the N-terminal amino acid residue, the half-life of the vaccine construct was calculated. The vaccine construct's in vitro stability was assessed, and an index value of ≤40.0 indicates that the input protein is stable. The total relative volume of aliphatic side chains was used to estimate the aliphatic index, which indicates the thermostability of the proteins. The grand average of hydropathicity index (GRAVY) value was determined by dividing the total hydropathy of all amino acids by the total amount of amino acids in the protein [21].

Allergenicity, antigenicity, and epitope screening

The protective antigens were predicted by the VaxiJen V 2.0 server with a threshold value of 0.5 (http://www.ddgpharmfac.net/vaxijen/VaxiJen/VaxiJen.html), which performs an autonomous alignment to predict the protective antigens. The accuracy of this server varies from 70% to 89%, depending on the organism [12]. To check the antigenicity of the final vaccine design, the construct was examined with ANTIGENPro (https://scratch.proteomics.ics.uci.edu/), a sequence-based, alignment-free, and pathogen-independent internet service. Prediction is a two-step procedure including five algorithms and several representations of the fundamental sequence [22].

The allergenic properties of the vaccine design were predicted using the AllerTop (http://www.ddg-pharmfac.net/AllerTOP/) server. It is an alignment-independent method that recognizes allergens based on the chemical makeup of amino acids and employs auto cross-covariance (ACC) to convert protein sequences into equal-length vectors. Using a training set of 2427 known allergens from various species and 2427 non-allergens, the proteins are classified using the k-nearest neighbor algorithm (kNN, k = 1) [23].

Prediction of secondary and tertiary structures, refinement, and validation

The designed vaccine contains a-helix, b-sheet, and coil/turns, which were analyzed by two online servers for secondary structure formation, i.e., PSIPRED v4.01 and Garnier-Osguthorpe-Robson (GOR IV) [24,25].

The template-based RaptorX server was used to derive the 3D structure of the vaccination protein in the next stage. The RaptorX server was also used to assess a number of projected structural properties, including disordered regions, binding sites, and solvent accessibility [26]. The GalaxyRefine server (https://galaxy.seoklab.org/cgi-bin/submit.cgi?type=REFINE) was then used to improve the quality of the created model structure. This server is based on the rebuilding of side chains, side-chain repacking, and eventually the relaxing of the structure using molecular dynamics [27]. For validation purposes, the derived models were evaluated using Ramachandran plot analysis and the PROCHECK service [28]. Moreover, the SAVES v5.0 server, which incorporates ERRAT, ProSA, and Verify-3D, was utilized for vaccine structural validation [29].

Disulfide engineering

Disulfide engineering was used to improve the protein's stability and geometric conformation using the web-based program Disulfide by Design 2.0 (DbD2, Wayne State University, Detroit, MI), which is found at http://cptweb.cpt.wayne.edu/DbD2/ [30]. In general, the DbD2 server finds the pair of residues in a protein structure that has a high possibility of generating disulfide bonds owing to cysteine mutation.

Molecular docking of the vaccine construct

The 3D structure of the TLR2 (PDB ID: 6NIG) was retrieved from PDB and prepared for docking study [31]. Molecular docking analysis was carried out using the Hdock server to comprehend the binding relationship between the improved model and receptor TLR2 [32]. A molecular dynamics (MD) simulation research was carried out on the complex that had the lowest docking energy and the highest binding affinity with the immunological receptor (TLR2).

Molecular dynamics simulations

The impact of the stability of the vaccine construct (TLR2-vaccine complex) was examined through the MD simulation process using GROMACS software along with the CHARMM36 force field. The complex was solvated within a cubic box of the transferable intermolecular potential with a three-point (TIP3P) water model while allowing a minimum of 10 Å marginal distance between the protein and each side of the 3D box. Under periodic boundary conditions implementation, the solvated system was neutralized by adding sufficient numbers of K+ and Cl− ions, followed by the steepest descent energy minimization algorithm. The subsequent step involved equilibration of the system under a constant network virtual terminal (NVT) and network time protocol (NTP) ensemble at 1 atm and 303.15 K. Finally, the MD simulation was run for 200 ns for 2 fs. After completion of the simulation run, the resulting MD trajectories were analyzed in terms of root mean square deviation (RMSD), root mean square fluctuation (RMSF), radius of gyration (Rg), and solvent-accessible surface area (SASA) by using the GROMACS built-in tools.

Codon optimization and in silico cloning

The expressibility of the vaccine construct in *Escherichia coli* cells was examined using the restriction cloning technique [33]. A crucial step for preserving the expression of the core vaccination sequence is optimization. Therefore, the Java Codon adaption tool (JCAT) (www.jcat.de/) was used to examine codon optimization and establish a link between gene expression and codon usage [34,35]. The vaccine sequence was used as input in this investigation, and the K12 strain of *E. coli* was chosen as the expression system. The values of the GC (guanine-cytosine) content, the codon adaptation index, and the length of the sequence were used to calculate the expression efficiency of the suggested vaccination. The pET28a vector and XhoI and BamHI restriction sites in the N- and C-terminal were chosen to carry out the restriction cloning.

## Results

Antigenic propensity

The ability of the individual proteins to produce an immunogenic reaction was selected on the basis of antigenicity score (Table 1). This prediction approach had a 75% accuracy rate. Each of the three proteins had an average antigenic propensity of >0.9, indicating that they are immunogenic.

**Table 1:** List of selected proteins used for vaccine development. PAP: predicted antigenic peptides; AD: antigenic determinants.

Unipro ID	Protein	Length	VaxiJen	PAP	AD
A0A0F3MFE6	Chaperone protein DnaJ	377	0.8022	1.0112	12
A0A2U3QNV5	30S ribosomal protein S12	123	0.8217	1.024	4
A0A0F3M9C7	Membrane protein (putative ompA-like autotransporter)	167	0.8010	1.0338	7

B-cell epitope prediction

Predicting B-cell epitopes is an essential step in the design of vaccines. They exist on the cell surface and are responsible for the secretion of antibodies that trigger the generation of the humoral or cellular immune response. B-cell epitopes were predicted using the ABCpred server, and a total of three epitopes for three proteins with the highest scores were chosen to create the subunit vaccine along with appropriate linkers and an adjuvant, which are shown in Table 2.

**Table 2:** B-cell epitope prediction for vaccine construction.

	Score	B-cell	Position	Allergenicity	Antigenicity	Score	ToxinPred
1	0.92	YGEGRYINTRNLEVKI	208	Non-allergen	Antigen	1.7987	Non-toxin
2	0.91	KSKIRASKSPALNGNP	13	Non-allergen	Antigen	0.8053	Non-toxin
3	0.84	VLSSPSICTANNNVNF	20	Non-allergen	Antigen	0.8470	Non-toxin

Prediction of HTL epitopes

Prediction of HTL epitopes is necessary for the development of both immunotherapeutic and preventive vaccines. Since T cells generate cytokines that control all adaptive immunological responses, they are crucial components of the adaptive immune system. They perform various kinds of functions, such as B-cell activation to create antibodies, B-cell class switching, macrophage activation for microbial elimination, and CTL activation to destroy infected target cells. By first detecting the peptide, major histocompatibility complex (MHC), HTLs indicate the activation of B-cells. They also help to release lymphokines, granulocyte-macrophage colony-stimulating factor (GM-CSF), and IFN, which improves both CTL and humoral immune responses. The IEDB web server was used to predict MHC-II-specific HTL epitopes for the alleles HLA-DPA1*02:01/DPB1*14:01 and HLA-DQA1*01:02/DQB1*06:02, which cover 81.81% of the global population and were chosen based on the geographical distribution of parasite-caused illness. The HTL epitopes were then sorted according to their IC50 value and percentile rank (Table 3).

**Table 3:** HTL epitopes prediction for vaccine construction. HTL: helper T lymphocytes; IFN-γ: interferon-gamma.

S. No.	Allele	Epitope	Method	Percentile rank	IFN-γ inducer	
					Result	Score
1	HLA-DRB1*11:01	QEEIKRAYRKLVLKY	Consensus	0.59	Positive	0.31272811
2	HLA-DRB3*02:02	GRKSKIRASKSPALN	Consensus	0.67	Positive	0.24791823
3	HLA-DRB3*01:01	GIGYRLDRHRMDVRL	Consensus	0.44	Positive	0.38684159

IFN-γ-inducing epitopes prediction

Upon activation by antigen-presenting cells (APCs), naïve T cells undergo differentiation into either Th1 or Th2 helper cells, subsequently leading to the secretion of cytokines. Interferon-gamma (IFN-γ) is classified as a type II interferon, which is secreted upon the differentiation of Th1 cells. During both innate and adaptive immune reactions, IFN-γ is made by Th1 cells. It activates macrophages, which are essential to treat intracellular infections. All of the epitopes studied were shown to stimulate IFN-γ production (Table 3).

CTL epitope prediction

The immune system is made up of several chemical compounds and cell types that cooperate to eliminate foreign substances while preserving cellular integrity. CTLs are CD8+ T cells that are able to recognize foreign antigen fragments on MHC-I molecules. They then release perforin, granzymes, or granulysin to kill target cells or bind Fas ligands to target cell Fas receptors to destroy them, protecting against both intracellular and extracellular pathogenic diseases. MHC-I-specific CTL epitopes for the three input protein sequences were obtained using the NetCTL 1.2 server. Three MHC-I supertypes, A2, A3, and B7, were chosen to cover 88.3% of the global population. For the three proteins belonging to the A2-, A3-, and B7-supertypes, a total of nine CTL epitopes were chosen (Table 4).

**Table 4:** CTL epitope prediction for vaccine construction. CTL: cytotoxic T lymphocytes.

S. No.	Epitope					
	A2	Score	A3	Score	B7	Score
1	YINTRNLEV	0.461	RAYRKLVLK	1.049	CPTCRGSGV	1.013
2	NLQEHSTVL	0.956	LVKTITPRK	0.652	TPRKPNSAL	0.687
3	SLLQPCYAV	0.506	TLEAGIGYR	1.2	RPLPIHIRF	2.44

Toxicity prediction of the selected epitopes

The toxicity of the relevant epitopes was assessed using the ToxinPred module. The three HTL epitopes, three B-cell epitopes, and nine CTL epitopes, all of which have been shown to be non-toxic in nature, were all used in the development of the vaccine candidate.

Designing multi-epitope subunit vaccine, physio-chemical characterization, immunogenicity, allergenicity, and solubility determination

The chosen BCL (3), CTL (9), and HTL (3) epitopes were joined by KK, AAY, and GPGPG linkers to form the vaccine. To offer the amino acid residues flexibility for protein folding, linkers were utilized. An adjuvant lipoprotein LprA agonist of TLR2 was added to the vaccine to enhance the vaccine-mediated immunological response. The final vaccine contains a 468 amino acid sequence. The suggested vaccine has a molecular weight of 49408.54 kDa and an isoelectric point (PI) of 9.76, according to the physicochemical evaluation. This hints at the protein's fundamental nature. Furthermore, the half-life in vertebrate reticulocytes, *E. coli*, and yeast was calculated to be >30, >20, and >10, respectively. The aliphatic index was determined to be 85.56, indicating that the construct is thermostable. The calculated instability index is 20.34, suggesting that the construct is stable. The construct would be hydrophobic based on the GRAVY score, which was found to be -0.224. The physicochemical parameters of the final vaccine design and positive controls were examined, and the suggested vaccine had nearly identical properties to the positive controls. Furthermore, according to the VaxiJen and ANTIGENPro servers, the antigenicity of the suggested vaccination was 0.7869 and 0.876720, respectively. The closest protein was found to be monofunctional C1-tetrahydrofolate synthase, mitochondrial (Q6UB35), which is a non-allergen and the protein shows immunogenic qualities. The AllerTop service predicts the allergenic nature of the vaccine. Furthermore, upon overexpression in the *E. coli* system, the vaccine design was expected to be soluble (0.702).

Structure prediction and analysis of vaccine construct

**Figure 1:**
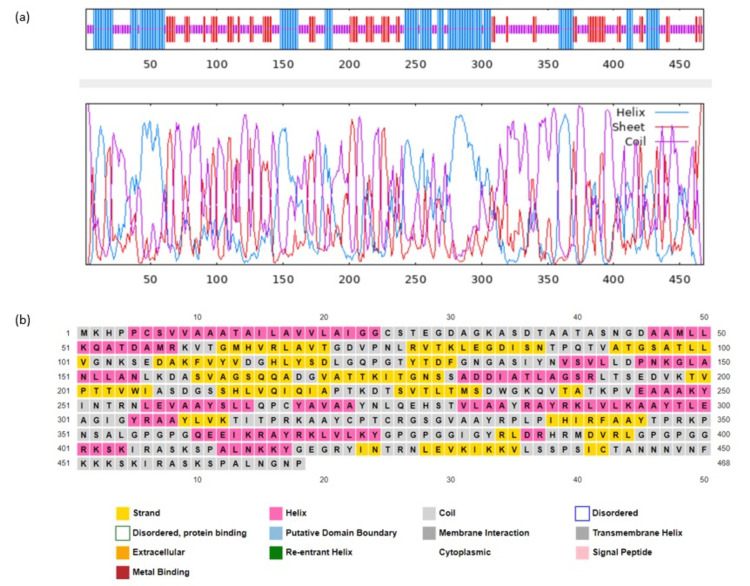
Secondary structure prediction of designed multi-epitope vaccine. (a) GOR IV server represented that the vaccine is comprised of a-helix (30.56%), coil (48.50%), and b-strand (20.94%). (b) Graphical representation of the secondary structure by PSIPRED server.

The secondary structure of the vaccine protein was predicted using the GOR IV and PSIPRED server to calculate the amino acid proportion of the a-helix, b-strand, and coil. The predicted composition of the vaccine includes 227 (48.50%) coil, 98 (20.94) b-strand, and 143 (30.56%) a-helix, respectively. A graphical representation of the secondary structure of the designed vaccine is provided in Figure 1.

**Figure 2:**
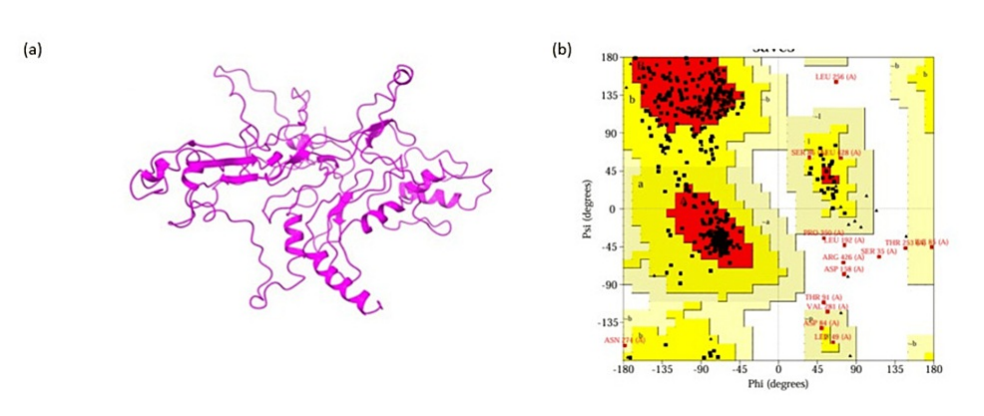
The diagram represents the final 3D structure of the subunit vaccine refinement. (a) The 3D structure of the constructed vaccine obtained by RaptorX server. (b) Graphical representation of Ramachandran plot obtained for the refined model.

The 3D structure of the vaccine was predicted by using the RaptorX server (Figure 2A). The disorderliness and p-value of the constructed vaccine were 8.40e-10 and 11%, respectively. Global distance test (GDT) and universal distance test (uGDT) values were 268 and 46, respectively. According to the findings on solvent accessibility, 22% of the residues are medium, 31% are buried, and 45% are exposed. The vaccine was made up of 30% helix, 20% b-sheet, and 48% loop. The Ramachandran plot statistics were used to validate the modeled structure on the PROCHECK server (Figure 2B).

Disulfide engineering

Disulfide engineering was used to increase the stability of the revised structure. There were 80 pairs of residues found that might be relevant for disulfide engineering. Three couples (Val19-Gly379, Glu245-Ala283, Val111-Asn178) were chosen based on their chi3 value (87 and 97, respectively), energy (1-2.2 kcal/mol), and B-factor (29-33).

Molecular docking

**Figure 3:**
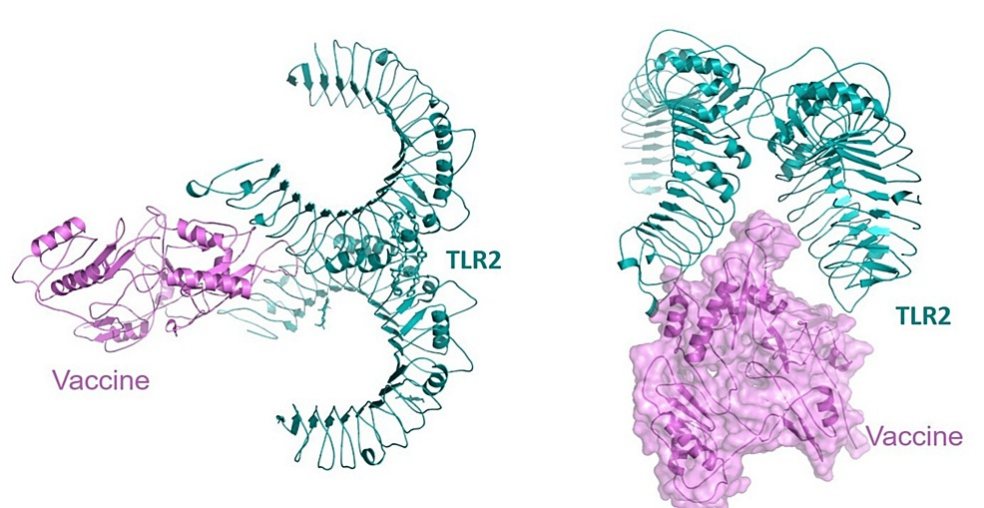
Molecular docking of vaccine construct with immune receptor. TLR2: toll-like receptor 2.

The immunological receptors TLR2 were docked with the vaccination design using the Hdock server. TLR2 has the lowest energy value of -309.70 kJ mol. Figure 3 depicts vaccine-receptor complexes. The docked complexes were then studied using MD modeling to determine their stability.

Molecular dynamic simulation

The 160 ns MD simulation research was used to investigate the binding mechanism, dynamic mobility, and stability of the receptor-vaccine cluster. The atomic level interaction between the vaccination protein and the TLR2 receptor complex was defined using GROMACS-2021, and the RMSD, RMSF, and hydrogen bonds were computed.

**Figure 4:**
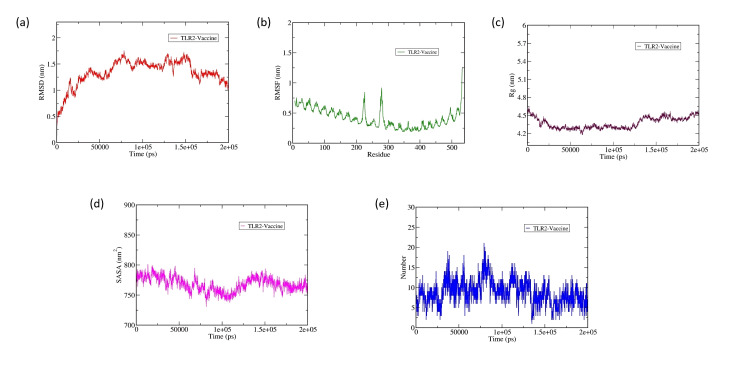
Molecular dynamics simulation results of the vaccine complexed with receptor (TLR2). (a) The root mean square deviation (RMSD) plot of the docked complex. (b) The root mean square fluctuation (RMSF) plot of the docked complex. (c) The radius of gyration (Rg) vs. time for the vaccine construct. (d) The solvent-accessible surface area (SASA) plot of the docked complex. (e) The hydrogen bond plot of the receptor-ligand complex depicts the specificity of intermolecular interactions.

The RMSD analysis of the TLR2-vaccine complex shows that the higher RMSD (~1.5 nm) of the complex is high due to the binding of the vaccine with the TLR2 protein. However, the complex tried to stabilize from ~50 ns to ~125 ns and it showed little fluctuation from ~125 to ~160 and again gained its stability without ~0.3 nm (Figure 4A).

The RMSF plot analysis has shown that higher fluctuation is observed at the C-terminal end of the TLR2 up to ~1.25 nm and the residues of b-sheet a-helix b-sheet containing regions of two TLR2 monomer-binding regions (Figure 4B). The gyration and SASA plots showed that the TLR2 and vaccine complex attained its compactness from ~25 ns to ~125th ns and lost its compactness from ~125 to ~160 ns and again gained its stability till the end of the simulation (Figures 4C, 4D). The inter-hydrogen bond plot shows that the TLR2 vaccine complex is stabilized by nearly 15 H-bonds, but has tried to maintain nearly 12 H-bonds throughout the MD production run (Figure 4E).

Overall, the RMSD, RMSF, gyration, SASA, and H-bond analysis plots showed that the complex showed stability from ~25th ns to ~125th ns and again started fluctuating and again from ~160 to ~200 ns it attained its stability. The fluctuation is mainly due to two reasons: (1) the reduced number of H-bond between the TLR2-vaccine complex is reduced from ~125 to ~160 ns; (2) the residues of b-sheet a-helix b-sheet containing regions of two TLR2 monomer interface regions.

In silico cloning into a microbial expression vector

**Figure 5:**
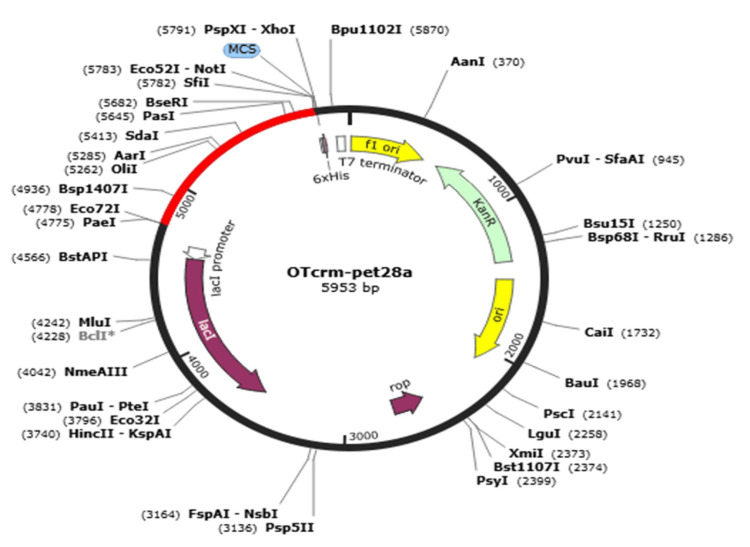
pET28a(+) expression vector with optimized codons for the vaccine protein inserted (red) in an in silico cloning map.

Codon optimization was used to clone the suggested vaccine design into an appropriate expression vector using an in silico method. Here, *E. coli* (K12 strain) was selected as the cloning host because it allows for greater expression in the microbial system. The construct's GC content was found to be 67.87% and its codon adaptation index was 0.54%. Furthermore, the restriction enzymes XhoI and BamHI were selected and put at the beginning and end of the sequence, respectively. Eventually, this approach produced a gene with a 1404 bp sequence length and a restriction clone with a sequence length of 5953 bp (Figure 5).

## Discussion

The development of an effective vaccine against scrub typhus caused by *Orientia tsutsugamushi* has been a longstanding challenge in the field of infectious diseases. The limitations of current therapeutic strategies, which primarily rely on antibiotics, highlight the urgent need for a safe and efficacious vaccine against scrub typhus [7]. One of the key challenges in vaccine development for this disease is the antigenic diversity of *Orientia tsutsugamushi* strains, which hampers the safety and efficacy of traditional whole-inactivated pathogen vaccine strategies [8]. To address this challenge, our study employs a multi-epitope subunit vaccine strategy, utilizing computational immunoinformatics tools to predict antigenic targets and design a vaccine construct.

Subunit vaccines comprising immunogenic epitopes offer several advantages over traditional live-attenuated vaccines, including safety, the ability to target specific antigens, and the potential for long-term protection particularly beneficial for individuals with weakened immune systems. By targeting multiple epitopes, these vaccines can stimulate both humoral and cell-mediated immune responses, providing broader protection against infectious agents [9]. In the case of scrub typhus, which exhibits antigenic diversity, a multi-epitope approach allows for the inclusion of conserved epitopes, potentially increasing vaccine efficacy across different strains of *O. tsutsugamushi*.

The construction of the proposed vaccine involved a meticulous process integrating computational immunoinformatics tools and structural biology techniques. The selection of antigenic proteins and epitopes was crucial to ensure the vaccine's efficacy in eliciting a robust immune response. By leveraging tools such as VaxiJen, ABCpred, and NetCTL, epitopes were accurately predicted, encompassing B-cell, CTL, and HTL epitopes, essential for inducing both humoral and cell-mediated immune responses. Moreover, the inclusion of an adjuvant further enhances the vaccine's immunogenicity, which is crucial for mounting a potent and durable immune response against *O. tsutsugamushi* [36].

B-cell epitopes play a crucial role in initiating the humoral immune response by eliciting the production of antibodies. The prediction of B-cell epitopes using computational tools such as the ABCpred server enabled the identification of potential targets for antibody recognition. By incorporating these epitopes into the vaccine construct, the immune system can generate antibodies capable of neutralizing the pathogen, thereby preventing infection [36,37]. Similarly, the prediction of T-cell epitopes, including both CTL and HTL epitopes, is essential for inducing cell-mediated immunity. CTL epitopes facilitate the recognition and elimination of infected cells, while HTL epitopes help orchestrate the immune response by activating other immune cells. The use of predictive algorithms to identify MHC-binding epitopes ensures compatibility with a broad range of human populations, enhancing vaccine coverage [36].

The final vaccine construct was designed to be immunogenic, stable, and efficiently expressible. The in silico analyses predicted that the vaccine possesses favorable physicochemical properties, including a high antigenicity score, a stable structure, and non-allergenicity. Moreover, the absence of allergenicity suggests a good safety profile [23]. Docking simulations with the TLR2 receptor, a key component of the immune system, indicated a stable interaction between the vaccine and the receptor, further supporting its potential efficacy. Disulfide engineering was employed to enhance the vaccine's stability, ensuring its integrity under physiological conditions. Finally, in silico cloning predicted efficient expression of the vaccine in *E. coli*, a commonly used expression system for recombinant proteins [38]. Codon optimization further enhances the vaccine's expressibility, paving the way for downstream experimental validation studies.

Limitations

Despite these advancements, it is essential to acknowledge certain limitations and challenges. Firstly, while computational tools provide valuable insights into vaccine design, experimental validation through in vitro and in vivo studies is imperative to confirm its efficacy and safety. Moreover, the complexity of host-pathogen interactions and the diverse genetic landscape of *O. tsutsugamushi* strains may influence vaccine efficacy and necessitate ongoing surveillance and optimization efforts.

## Conclusions

The development of a multi-epitope subunit vaccine against *Orientia tsutsugamushi* represents a promising strategy in the fight against scrub typhus. Through a comprehensive computational approach, the proposed vaccine was discovered to be highly antigenic, stable, and non-allergenic, laying the foundation for further experimental validation and clinical translation. Furthermore, the presence of B-cell, CTL, and HTL epitopes indicates that this vaccine may be capable of inducing both humoral and cell-mediated immunity in humans. However, the proposed vaccine needs to undergo in vitro and in vivo validation to prove its efficacy against the disease. Continued research and collaborative efforts are warranted to address remaining challenges and expedite the development of an effective vaccine against this debilitating infectious disease.
